# Systems analysis of the effects of the 2014-16 Ebola crisis on WHO-reporting nations’ policy adaptations and 2020-21 COVID-19 response: a systematized review

**DOI:** 10.1186/s12992-023-00997-8

**Published:** 2023-12-05

**Authors:** Jessi Hanson-DeFusco, Min Shi, Zoe Du, Ornheilia Zounon, Fidèle Marc Hounnouvi, Albert DeFusco

**Affiliations:** 1 University of Texas at Dallas, Cecil H. Green Hall 3.526, 800 West Campbell Road, Richardson, TX 75080-3021 USA; 2 University of Texas at Dallas, Richardson, TX USA; 3 Université d’Abomey-Calavi, Cotonou, Benin; 4 Anaconda, Inc, University of Pittsburgh, Pittsburgh, USA

**Keywords:** Infectious-disease response, Ebola, COVID-19, Conditioned learning, Global health policy, Mixed-method analysis, Preparedness and prevention, Systems analysis, Systematized review

## Abstract

**Background:**

Recent case studies indicate that the 2014-2016 Ebola outbreak, one of the worst pre-2020 global biological catastrophes in modern history, helped some nations to better prepared their responses for the COVID-19 pandemic. While such national case studies explore how specific nations applied EVD-related policies in their domestic battle against the COVID-19 pandemic, there is no known study that assesses how many WHO nations learned from the West African crisis and to what scale.

**Objective:**

Applying the policy legacies analytical framework and a systematized literature review, this research examines how prior policy experiences with the 2014-16 EVD crisis as a large-scale emergent outbreak helped to inform and to condition WHO nations to proactively prepare their national policies and health systems for future threats, including ultimately COVID-19.

**Methods:**

A systematized literature review of 803 evaluated sources assesses to what extent Ebola-affected and non-affected nations directly modified governmental health systems in relation to this warning. The study further evaluates how nations with documented Ebola-related changes fared during COVID-19 compared to nations that did not. We present a categorical theoretical framework that allows for classifying different types of national response activities (termed *conditioned learning*).

**Results:**

Ten (90.9%) of 11 nations that were affected by 2014-16 Ebola crisis have documented evidence of repurposing their EVD-related policies to fight COVID-19. 164 (70.0%) of 234 non-EVD-affected nations had documented evidence of specifically adapting national systems to incorporate policy recommendations developed from the 2014-16 crisis, which informed their COVID-19 responses in 2020.

**Conclusions:**

The shock of 2014-16 EVD outbreak affected most nations around the world, whether they experienced Ebola cases. We further develop a categorical framework that helps characterised nations previous experiences with this biological catastrophe, providing a means to analyse to what extent that individual nations learned and how these EVD-related changes helped inform their COVID-19 response. Nations that demonstrated EVD-related conditioned learning nations tended to have more stringent COVID-19 responses before April 2020 and utilized documented response mechanisms developed out of the West African crisis.

## Introduction

Before the era of COVID, the 2014-16 Ebola outbreak in West Africa was considered one of the worst global biological catastrophes in modern history [1–5]. After 2016 when the West African crisis ended, various international health agencies including the World Health Organization (WHO) warned all national governments, even those not directly affected by Ebola, to strategically learn from the 2014-16 crisis to better prepare for *eminent future outbreaks* [2, 6–10]. This transcontinental epidemic prompted the largest and most costly global health response in generations [2, 4, 11–14].

Researchers are only recently beginning to understand the extent of the policy ramifications from the West African epidemic on national health preparedness and response prior to the COVID-19 pandemic [15–19]. A growing number of national case studies indicate that some countries like the governments of Ghana [20] and Nigeria [21, 22], took this recommendation seriously, and that their Ebola-related policy reforms later impacted their national responses to COVID [2, 3, 6, 7, 12]. However, there is no identified study to date that specifically measures the extent to which WHO nations incorporated Ebola-related policies in relation their COVID-19 preparedness and response.

Our study seeks to address this comparative gap in the literature by identifying how many nations adopted EVD-related policies prior to 2020. We perform a mixed method analysis of data collected from a systematized literature review of 803 documents. This study theoretically applies the *policy legacies analytical framework*, which is rooted to historical institutionalism (HI) literature examining the process of punctuated policy reforms through analysing the historical context and the eventual sequence of events that can influence political outcomes. A nation’s policy legacy can influence future policy learning [20, 23]. We further consider how the 2014-16 EVD outbreak acted as what Fligstein & McAdam (2012)’s systems approach refers to as an *exogenous shock* [24]. This specific catastrophic biological disaster was a systemic shock that caused seismic systemic changes in both affected and non-affected countries with policy legacies still yet to be explored. Health systems continually learn and build on experiences of global outbreaks [3, 18, 25, 26]. This learning is a kind of institutional memory that can be shared among affected and non-affected populations (termed conditioned learning) [8, 12]. Yet the quality of policy learning can vary [5, 8, 27].

### Purpose of this study

This study examines the following questions: *(1) How many WHO nations have documented evidence of integrating policies from the 2014-16 West African Ebola crisis to improve governmental health preparedness and response mechanisms before 2020?* *(2)* *What are key trends in variations in how they incorporated EVD-related modifications into their COVID responses?* Our intent is to consider the historical effects that EVD-related policies may have had, better determine how many nations may have incorporated national policies and reforms to their health systems as a direct result of the 2014-16 crisis, and to assess for patterns of *conditioned policy learning*. We initially hypothesize that the impact of the 2014-16 EVD outbreak in West Africa, which garnered extensive global media attention and had massive political-economic repercussions, directly prompted most nations to act (measured by qualitative documented evidence in official agency reports, studies, and relevant media).

In the next section, we present the methodology, including the theoretical framework, search strategy, key variables assessed, and data analysis of the systematized review. The rest of this article is presented as follows: the Results section summarizes the quantitative findings from the review, including how many nations incorporate EVD-related policies during 2014-2019. We additionally discuss key trends (categorized by common parameters of conditioned policy learning) that are qualitatively noted among many nations drawing from the PLAF. The next subsections each offer a more detailed quantitative analysis of nations falling within one of these conditioned learning categories. We conclude the paper by presenting research implications and limitations, along with proposed recommendations for future studies.

## Material and methods

### Theoretical framework

Our study applies the PLAF to understanding how two outbreak preparedness approaches can overlap because of the government’s historical handling of biological threats. Anti-Boasiako et al. (2023) find that a major causal factor in Ghana’s COVID-19 preparedness and response built on policy legacies from the West African Ebola crisis, mainly lessons learnt that were applied after Ebola outbroke in sister nations [20]. PLAF is theoretically linked to historical institutionalism (HI), which postulates that history indeed matters in policy science. History often offers a contextual foundation and cognitive foundation that explain how current policy choices are reached [20, 28–30]. A current policy is more likely informed by the consequences of relevant past policymaking than socio-economic factors. Policymakers’ actions often are informed by *policy legacies*, or purposeful reactions to previous policies [20, 31–33]. Established institutions and their policy legacies can either limit policy change, or can create chances for positive reform [20, 28, 33].

As a mechanism of path dependency, successful past policies can influence policymakers’ preferences to select similar current policies as problem solutions [23]. There are several critiques of whether policy legacies allow for policy learning [23, 34, 35]. Nair & Howlett (2016) posit that some policy legacies can inform and establish institutional routines and procedures that can be stifling for decisionmakers [36]. However, when past policies fail, and new policies arise out of a critical need for innovation, this can inspire policymakers to learn [23, 34, 35]. Additionally, policy learning can also happen when policymakers learn from successful past policies [23].

### Search strategy

The three primary authors conducted a systematized review (May 2021-January 2023) of secondary data sources, mainly peer-reviewed studies, governmental reports and documents, reports from international organizations like the WHO and Center for Disease Control (CDC), and electronic media reports. The method of systematized reviews is increasingly used in global health policy [37, 38]. We utilized elements of PRISMA reporting guidelines including applying protocols and stages that guide filtering and synthesizing large bodies of documented evidence related to the research question [39]. By incorporating various elements of standardized method involving pre-selected eligibility and exclusion criteria, researchers may reduce the risks of selection biases of relevant sources [37, 38]. Literature reviews are more susceptible to selecting data sources related to each other. Comparatively, systematized literature reviews allow more space for additional interdisciplinary sources to also be considered in the review process, widening the body of information and thus the understanding of the problem at hand [2, 4, 40].

**Fig. 1:**
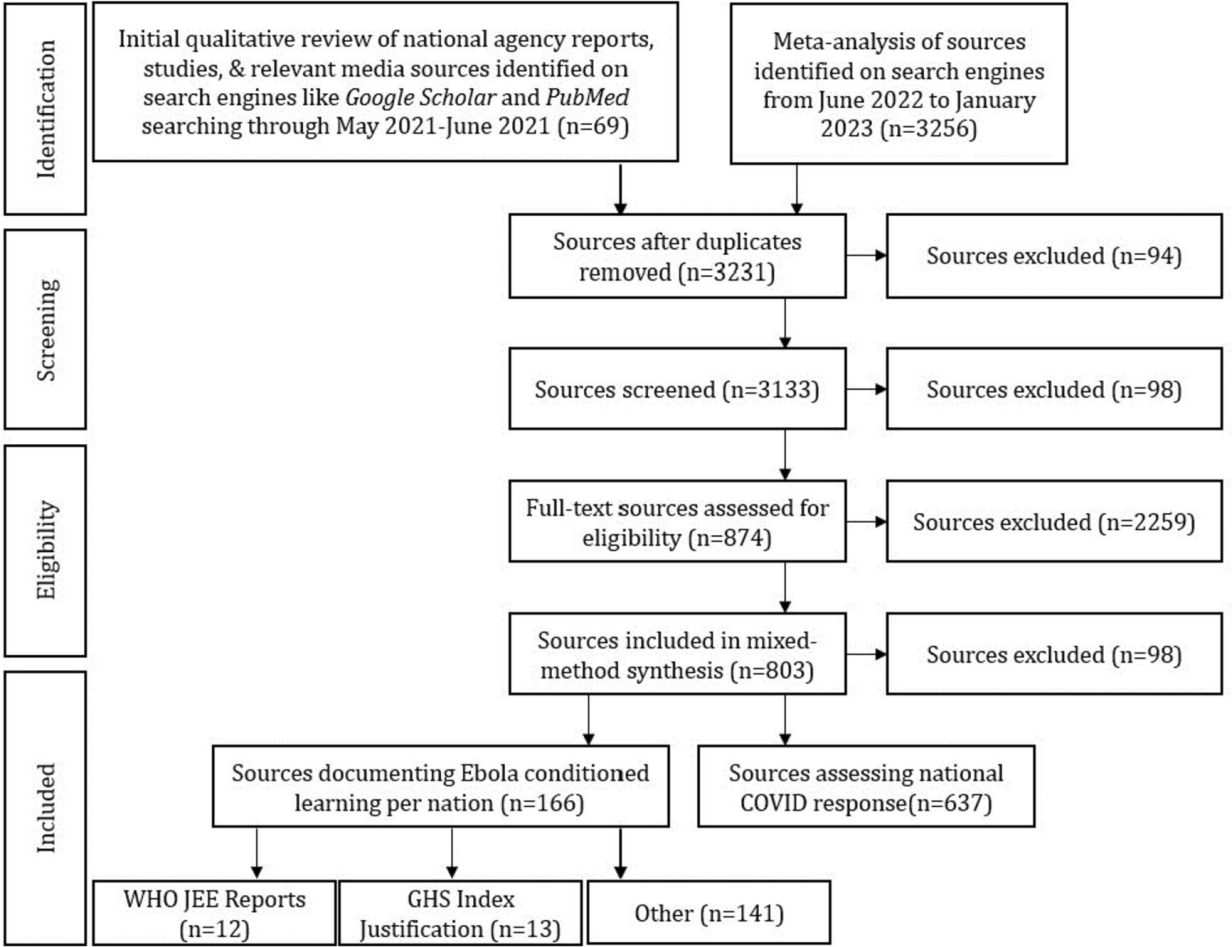
Systematized review process

The search criteria conducted on key search engines including Google Scholar and PubMed included search terms of *Ebola, West Africa, COVID-19, health system, policy reform, prevention, changes,* and each nation’s name. In the screening process, reviewers independently researched specific national cases one-by-one. Top-cited studies were prioritized. The synthesis also included grey literature of formal documents from governmental and ministerial websites and relevant digital media sites to summarizing the response activity of each country at the start of the COVID-19 outbreak, as well as formal health and government documents. Overall, 3231 sources were screened in the first round (see Fig. 1).

Each investigator assessed each data source based on specified eligibility criteria: (a) written mainly in English, or if in another language, could be legibly translatable through Google Scholar for interpretation; (b) mentions EVD and/or COVID-19; and (c) was published after 2014. Each selected source was then again reviewed by another investigator to validate its eligibility. 169 sources were excluded as irrelevant (in a language with a poor English translation making it difficult to understand; not directly related to research topic; referring to EVD but before the 2014-15 outbreak; or being a duplicate). In cases of discrepancy, the PI had final determination of whether to include the document as eligible.

### Data analysis

In the data analysis phase, we examined each country individually to assess if there was any documented evidence of the nation incorporating EVD-related policies during 2014-2016, against whether it directly experienced the West African crisis, and if there was mention of these modifications being applied in 2020 (see Table 1). In total, 803 sources of those considered were included in the final analysis. A double-review was later conducted to minimize potential subjective biases in the categorization process [41, 42]. In October 2022-January 2023, each country analysis was secondarily reviewed by another investigator from our team for concurrence. As part of an initial sensitivity analysis, we prioritize evidence that came from peer-reviewed studies, government documents, and official agency reports. Media documentation was also used in the analysis, including triangulating research and government documentary evidence.

**Table 1:**
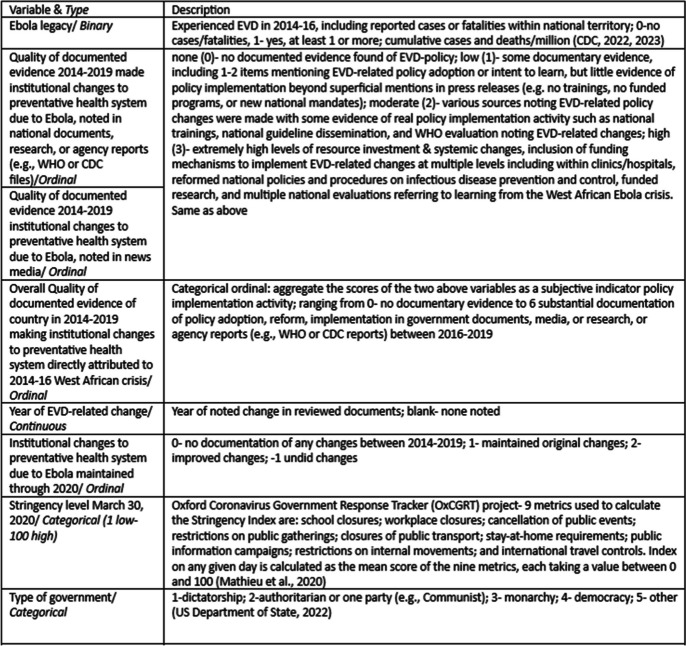
Study variables

In January 2023, three additional investigators were invited by our team to support the data analysis, discussion, and comparative case studies presented in the Results. Firstly, we aggregated the number of WHO countries that have some evidence of incorporating some level of EVD-related policies into their national systems during 2014-2019. Lastly, the entire research team reviewed, discussed, and determined through a series of analyses key trends in how nations experienced EVD-related policy legacies in their national COVID-19 preparedness & response mechanisms.

We present the overall results in the next section, including how many countries appear to have incorporated EVD-related policy changes and how. Secondly, we present the key trends of how countries applied EVD-related policies directly to their individual COVID responses learning categories. We present the disaggregated total of how many countries fall within each category, and compare national case studies to exemplify these trends through the policy legacy analytical approach.

## Results

The systematized review indicates that of the 11 nations affected by the 2014-16 Ebola crisis (Spain, Italy, Liberia, Sierra Leone, Guinea, Mali, Nigeria, Democratic Republic of the Congo (DRC)^1^ USA, Senegal, and the United Kingdom), nearly all maintained EVD-related policies for response and prevention, which in turn informed their COVID responses in 2020. In comparison, 164 (70.00%) of 234 non-EVD-affected nations had documented evidence of specifically adapting national systems to incorporate policy recommendations developed from the 2014-16 crisis. These nations tended to perform better in their response information management, outbreak containment in the first waves of COVID, more rapid and stringent policymaking, and higher rates of population compliance and mandate enforcement.

While most nations incorporated EVD-policies before 2020, they had no significant difference in 2020 cumulative COVID cases/million (t = -0.93, *p* > 0.05) or deaths/million (t = -0.76, *p* > 0.05), as COVID overwhelmed most national preparedness systems as the pandemic raged on. Our review found evidence of these changes in governmental documents, research, and health agency reports for 157 nations (64.01%). Comparatively, we uncovered media evidence for 108 countries (44.08%) reporting governmental public health reforms in direct response to the West African crisis. Secondary sources including media documenting these same changes triangulate and validate findings in official documents and research reports [42, 45, 46]. Yet it also indicates that while most nations made EVD-related changes before COVID, these reforms may not have been readily publicized, as reflected by the lack of media coverage. Whether past policies failed or were successful, policy legacies can help the policy learning process [20, 23, 35].

Table 2 summarizes the level of evidence of EVD-related policy activity, from no evidence to high among the three main groups of policy legacy & learning. The first group is comprised of countries that were not directly affected by the 2014-16 outbreak (no deaths or cases), and appear to have no evidence in the literature of any adoption of EVD-related reforms before 2020. The second group are non-EVD-affected nations with some level of documented policy changes, most of which indicates that most of these countries adopted EVD-related policies, and conducted moderate policy activities like agency trainings, WHO technical consultations, and some protocol reforms and governmental resource allocation. The third policy legacy group is nations with EVD cases and deaths, most of which additionally demonstrated moderate policy activity, while a handful have strong policy reforms at the national systemic level (triangulated by media sources).

**Table 2:**
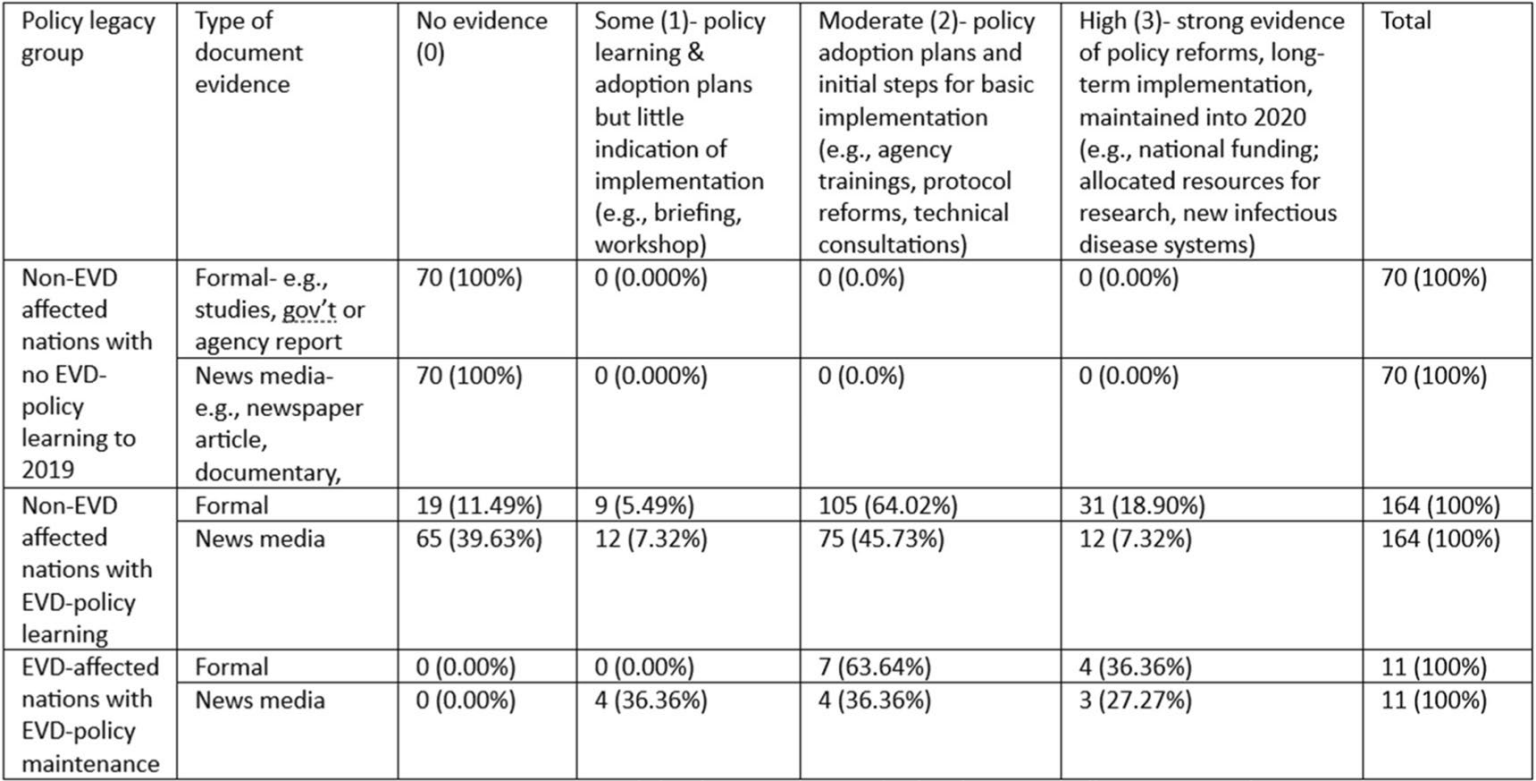
Disaggregation of documented evidence level of policy legacy grouping

Percentage in brackets; Formal documents- Pearson chi2= 1750.20, *p*=0.000; News media- chi2=103.09, *p*=0.000

The policy legacy of the 2014-16 EVD crisis may have been unique as a transcontinental biological threat that helped propel both international agencies and most national governments to take the threat of an imminent global outbreak more seriously [21, 47–51]. The systematic review indicates that many governments adopted and maintained public health policies developed out of the West African crisis, many of which were applied, modified, or repurposed for national COVID preparedness and response efforts. While institutions and decisionmakers may prefer maintaining the status quo, “there can be path departures in the absence of critical junctures since change can be exogenously spurred by cataclysmic events” [28].

### Variation in policy legacies and learning

**Fig. 2:**
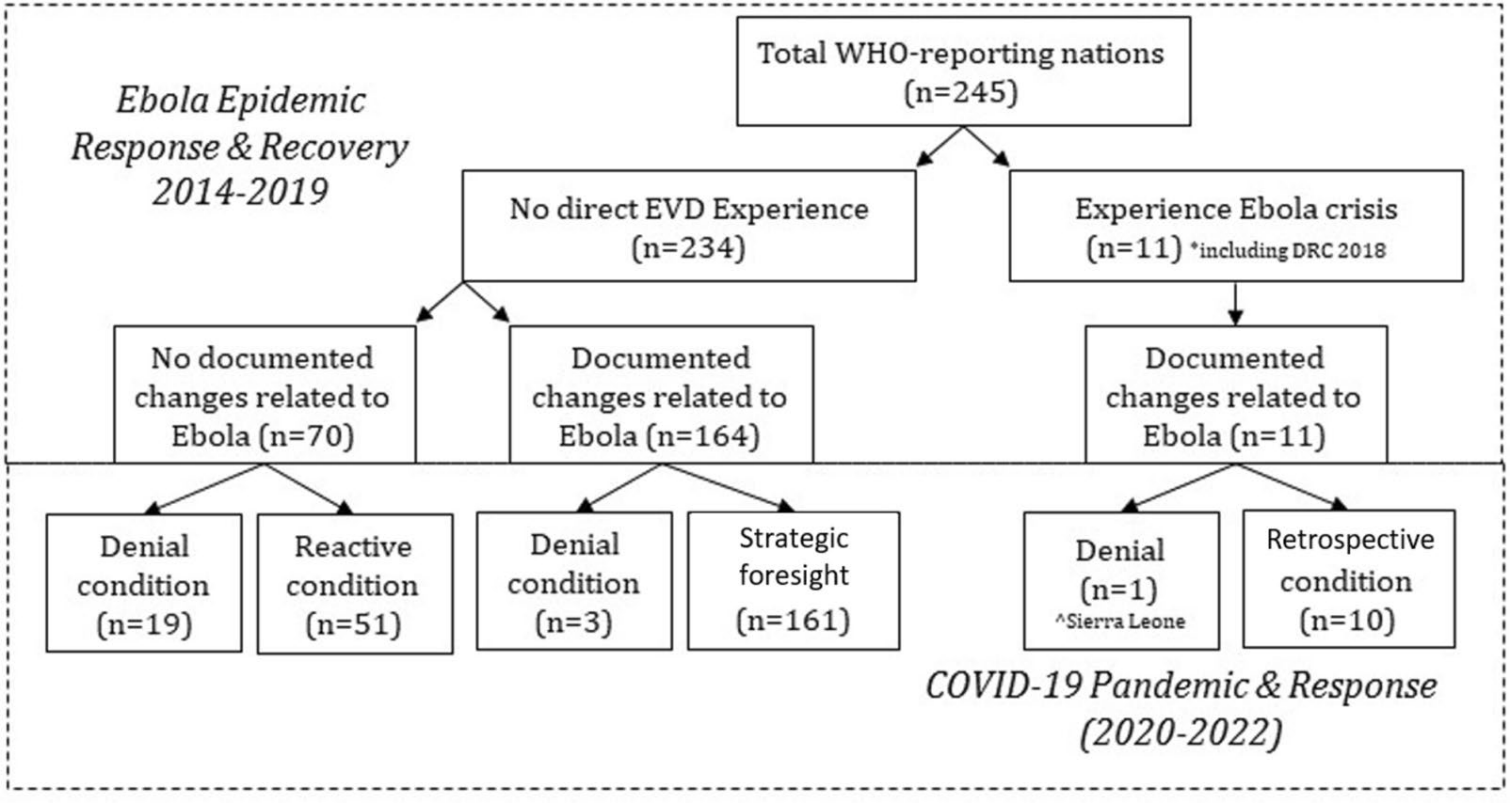
Conditioned policy learning of 2014-15 Ebola policies on WHO nations & COVID response

Our analysis identifies four key variations in how countries incorporated EVD-related policies, which we categorize as: *denial, reactive, strategic foresight,* and *retrospective learning*. These categories help us discern between types of learning experiences and quality of responses, and map common country policy legacy trends against one another [52]. Figure 2 depicts the disaggregation of policy learning trends (called conditioned policy learning) and how the 245 WHO nations eventually fall into one of the four categories in relation to their 2020 COVID responses.

Most countries that experienced the 2014-16 crisis firsthand (Ebola cases and/or deaths) maintained their policies, infrastructure, and public health systems adapted during EVD when COVID struck, reflecting *retrospective conditioning*. Comparatively, our analysis notes that some countries (both EVD-affected and non-affected) appear to have lingered in the condition of *denial*. Additionally, some countries that did not directly experience the West African EVD crisis remained in a state of denial when COVID first struck, and at first demonstrated hardly any policy response action. They did not have stringent COVID policies in the first months of 2020, and did not have a national-level shutdown typically until after April 1, 2020 (weeks after the WHO declared a state of emergency).

Comparatively, other non-EVD affected nations that had no documented EVD-related policy changes before 2020 quickly took prompt action to COVID in March 2020, but often in a chaotic manner (*reactive conditioning*). Their stringency levels of COVID mandates fluctuated throughout the spring and into summer. However, most non-EVD-affected nations applied EVD-related policies from the West African crisis, often recommended by the WHO and CDC, in the years following the outbreak. When COVID struck, these countries purposefully applied the EVD-related policy modifications in their COVID response plans (*a condition of strategic foresight*). The next section elaborates on these variations in policy legacies and policy learning.

### Countries that retrospectively learned

All nations that experienced *at least one EVD case* in 2014-16 had documented evidence of proactively adapting their health systems based on Ebola-related research, policy reform, and infrastructure and funding changes (measured by official government documents, agency documents, and/or media coverage). Yet, only ten of the 11 EVD-affected nations were able to *retrospectively* prepare and respond to COVID-19, meaning that they reactivated EVD-related policies when COVID first hit in 2020.

Reviewed governmental and health agency documents specify that these ten countries were frequently reapplying or modifying their Ebola-response mechanisms (including community engagement, case tracing, rapid testing, and quarantining procedures) to respond to COVID. This transition was frequently marked by quick reaction by the government, a stronger ability to mobilize preventative actions that signal containment of the disease, instead of inconsistent and cumbersome policy decisions with limited impact.

By the end of March 2020, stringency levels peaked worldwide with most nations locking-down before April. On March 31, 2020, the average stringency (low 1-100 high) in the 11 EVD-affected countries was quite high at 77.10 (SE = 3.41, CI95% 69.49-84.72). EVD-affected nations able to flatten the curve of the first COVID-19 pandemic wave tended to benefit from proactive planning on parts of national governments and international health stakeholders [50, 52–54]. As COVID struck in early 2020, these ten countries took decisive action to build on the health systems, infrastructure, and mechanisms put into place during Ebola.

**Fig. 3:**
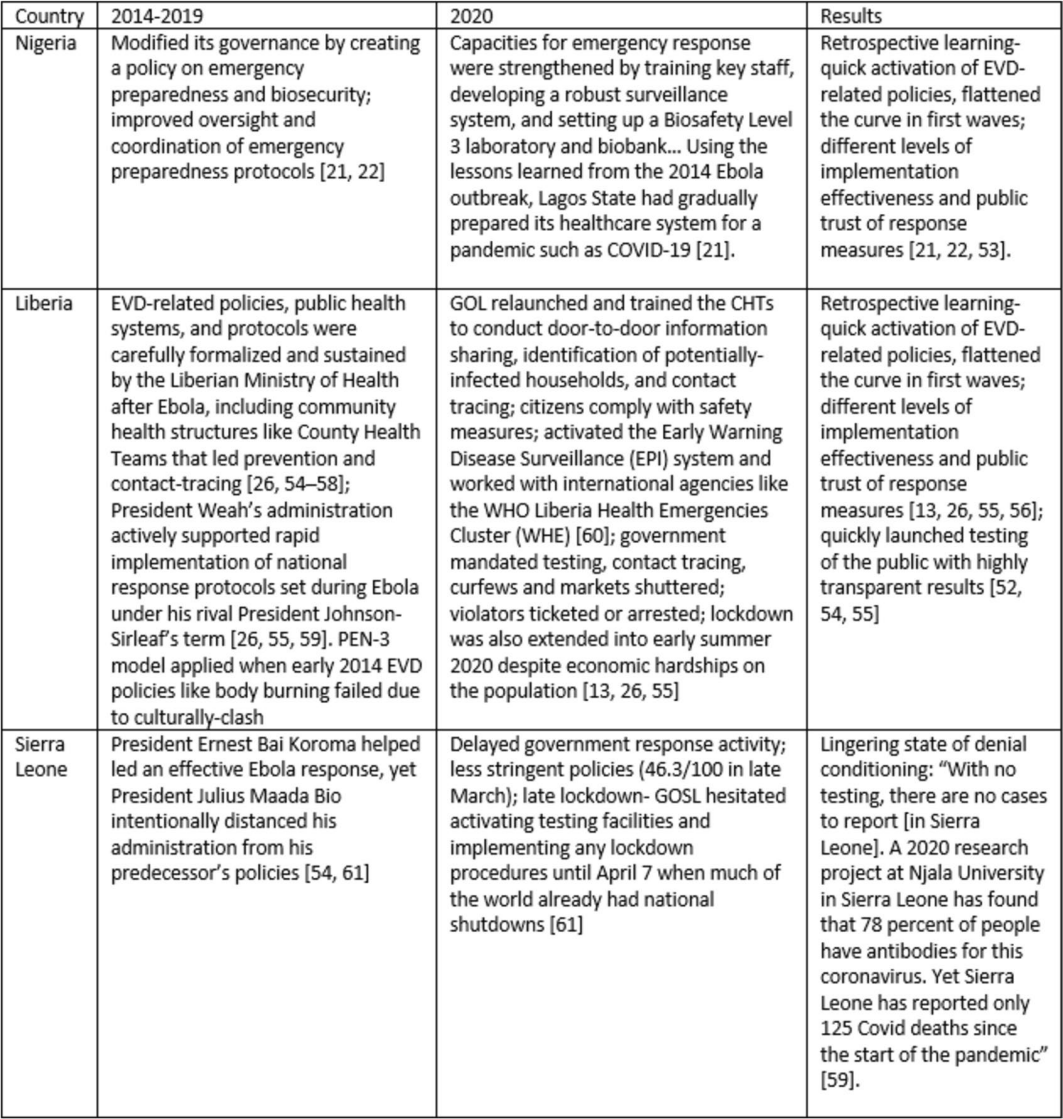
Case studies of EVD-affected countries & COVID-19 conditioned learning

Figure 3 provides case examples of EVD-related countries’ policy learning. Nigeria and Liberia’s COVID-19 national response in 2020 were notably organized and effective at mitigating the first waves often attributed to their EVD-related policy improvements [21, 22, 55–58]. One critical factor repeatedly noted is how many of these nations employed a key lesson learnt in the Ebola crisis- the need to identify culturally and contextually appropriate solutions [13, 21, 49, 51, 59]. Some initial Ebola policies promoted by international agencies, like body cremation, proved ineffectual, clashing with cultural beliefs and practices. Changes were eventually made to incorporate culturally-sensitive solutions proposed by West African experts [5, 11, 48, 59, 60]. Our health community was reminded of the importance in applying robust frameworks like the PEN-3 model, which are designed to guide global health experts and practitioners to carefully listen to cultural communities. We must first focus on the best practices of localized populations instead of starting with what global health policy dictates as wrong. “[S] ocietal reasoning and rationale are at the foundation of the message… reframing COVID-19 communication messages globally must respond not only to individuals but to the community as a collective” [47].

A second key factor appears to be in national leadership determining to maintain EVD-related policies even after large shifts in political power such as after an election. We can view this in the 2020 decision by President Weah’s administration to actively support rapid implementation of national response protocols set during Ebola under his rival President Johnson-Sirleaf’s term in office [25, 57, 61]. The effectiveness of Liberia’s 2020 COVID response under Weah’s administration has been compared to the responses of China, New Zealand, and Finland [56, 57, 62, 63].

In contrast, Sierra Leone appears the only 2014-16 EVD-affected country to remain in a temporal state of *denial* during early 2020 as COVID spread. Evidence indicates delayed government response activity, less stringent policies (46.3/100 in late March), and a late lockdown. Several EVD-affected nations like Sierra Leone and Liberia reported low COVID infection rates in 2020. However, Sierra Leone’s low infection rate is widely debated within the health community. Despite its high EVD rates in 2014-16, COVID testing among Sierra Leone’s population remained effectively non-existent through 2022. Furthermore, a 2018 political shift led to the undoing of many EVD measures under the former administrative leadership [61, 64, 65].

### Strategic foresight conditioned learning

Although they were not directly impacted by the West African crisis, the review found that 164 (70.00%) of the 234 non-EVD-affected nations had documented evidence of 2014-2019 policy modifications directly attributed to this specific epidemic. These modifications often were based on WHO-related recommendations developed from the West African crisis. Comparatively, 161 nations exhibit what we term *strategic foresight (SF) conditioning* by rapidly activating EVD-related system modifications by early March 2020 to combat COVID. Comparatively, three nations instead exhibit delayed *denial* responses (little COVID-response until April). The average end of March stringency level of SF nations was 75.35 (SE = 1.41, CI95% 72.61-78.16), with no significant difference compared to EVD-affected nations (t = -0.31, *p* = 0.62).

Our analysis indicates that often SF nations received guidance by lead international health organizations. The World Bank’s International Development Association-supported Regional Disease Surveillance Systems Enhancement (REDISSE) Program was launched in 2016 to support 2014-16 EVD-affected nations and non-affected nations (Benin, Burkina Faso, Cabo Verde, Cote d’Ivoire, The Gambia, Ghana, Guinea-Bissau, Mali, Mauritania, Niger, Nigeria, Senegal, Togo) to incorporate EVD-related policies to better guard against future epidemics. To varying levels of degree, these countries included REDISSE health reforms to combat COVID, including rapid laboratory testing, forming a technical preparedness working group, and activating Incident Action Plans transnationally [22, 66–68].

Additionally, after the West African Ebola crisis, nations performed self-assessments of their preparedness systems using a WHO checklist as part of the International Health Regulations Core Capacity Monitoring Framework. Moreover, technical missions were performed in 27 Latin American and Caribbean nations like the Bahamas to identify potential EVD cases within their territory. This required working in partnership with global health policy experts from national agencies and key national agencies [69]. Most of these countries activated elements of the IHR training in 2020. Yet even with this training, some nations like the British Virgin Islands remained in a brief state of *denial*, with no documented evidence that the nation learned from Ebola, and furthermore, greatly delayed in responding to COVID-19, with concerns of economic fallouts [70, 71].

### Reactive conditioned learning

Comparatively, our review did not reveal any documented evidence for 70 nations of incorporating EVD-related changes prior to 2020, of which 19 remained in a state of *denial* when COVID struck. Comparatively, 51 demonstrated *reactive conditioning*. For instance, the Bulgarian Parliament unanimously passed a declaration a state of emergency, with a 14-day preventive house quarantine for citizens traced to contact with a COVID-19 patient or a highly-infectious nation [72]. Reactive conditioning was often chaotic, but at times, some nations were able to react efficiently and effectively, provided they had proper resourcing and expertise. Overall, these 70 nations had a modestly lower average stringency rate around the end of March 2020 of 72.75 (SE = 3.61, CI95% 65.43-80.06), just as COVID began to first peak worldwide. Research indicates that the impact of the timing and level of mitigation policies in the spring of 2020 were generally important for lowering mortality from COVID-19 [61, 73, 74].

## Discussion

After the West African EVD crisis, health policy research increasingly advised the international community and national governments to carefully learn from the West African Ebola crisis [2, 8, 25]. Bill Gates stated, “Perhaps the only good news from the tragic Ebola epidemic in Guinea, Sierra Leone, and Liberia is that it may serve as a wake-up call: we must prepare for future epidemics of diseases that may spread more effectively than Ebola” [2]. Documenting evidence of how many nations used the West African crisis to inform policy change is a good indication of policy legacies linked to research utilization and policy learning, which can improve future policy effectualness [45, 61, 74, 75].

The literature reviewed for this study verifies that as early as 2016, the devastating West African Ebola emergency prompted major changes in how most national systems around the globe pre-emptively prepare for future outbreaks. The 2014-16 EVD crisis appears to have disrupted how modern political institutions collaborate with the health sector to prioritize preventing biological disasters [9, 15, 48, 76]. As a result of EVD, the WHO’s emergency response structure was altered to include operational capabilities to its traditional normative and technical roles, and the WHO Research and Development Blueprint initiative was launched to support rapid activation of R&D activities in an epidemic. Moreover, the WHO and CDC ramped up their partnerships with international agencies and national governments to improved rapid response funding mechanisms and testing [5, 8, 27].

Health systems continually build on experiences of global outbreaks. This research implies that the West African crisis appears to have acted as an exogenous shock for EVD-affected and non-affected nations. The changes caused by the 2014-16 EVD crisis were for the most part punctuated with long-term national-level impacts worldwide. By examining the historical experiences of nations’ COVID-19 preparedness & responses to policy legacies from the 2014-16 West African EVD crisis, we can interpret patterns that help us understand how a nation’s previous experience with a disease can condition not only its learning for future biological threats, but many other nations around the world. As a biological threat (EVD or COVID) begins to impact people within a territory, some countries can enter what this study terms- *denial conditioning*, a phase in which at least temporarily national agencies and leaders pay little attention or ignore the growing effects of the epidemic. But as an outbreak increasingly threatens the national population, the government sooner or later enters *reactive condition*, a phase focused on responding to the shock as if its effects will have minimal impact or be over quickly. After a nation has experienced an initial biological exogenous shock, it is likely to *retrospectively* draw on what it learned in future outbreaks. Its policy legacies can also motivate non-affected countries to proactively learn often by adopting policies related to its best practices and lessons learnt (*RF conditioning*). In these cases, leaders tended to provide ample additional health funding, resources, and personnel to improve preparedness strategies, tied directly to what the WHO and CDC learned from EVD.

Political windfalls including issues of transparency and undoing EVD-related policies established by political rivals can have massive impacts a nation’s capacity and the motivations behind a country’s response to a major outbreak like COVID. Bureaucratic institutions are influenced by political representatives who act as decisionmakers. Some representatives may lack critical information or may be influenced by political interests and support for once-effective policy legacies which later are irrelevant and impede response quality, the effects of which can carry over into public response activity [3, 7, 51].

As Ellermann (2015) explores, HI theorists debate whether policy legacies constrain policy learning [23]. We reposition this critique to focus on the historical intention(s) of policymakers to learn. In cases where decisionmakers in the country historically deny a public threat, like the growing prevalence of emerging outbreaks, they may be more likely to continue denying the problem exists even when it strikes their territory. For instance, Belarus had no documented evidence of learning from Ebola. Even after COVID struck, it remained one of the worst denial offenders with extremely low stringency levels in the first months of 2020 [77]. This study exemplifies how global health research can theoretically model conditioned policy learning.

## Limitations

Systematized reviews often have a greater likelihood of bias than studies strictly following guidelines of systematic reviews [38, 78]. Future research would improve the quality of the analysis by more extensively adhering to PRISMA guidelines, including extending the search strategy to include more search engines; conducting a more robust sensitivity analysis; and more effectively track studies that might have met our study inclusion criteria but were excluded [39, 78]. Additionally, the theoretical model does not fully account for conditions of mutual learning between countries (those who experienced the first wave of COVID-19, compared to those who experienced it during the second or third wave), nor the support of external resources such as scientific circles. For instance, Benin treatment protocol incorporated recommendations from French medical institutes and support from the World Bank [79].

Since the 2005 updates to the International Health Regulations (IHR), the WHO Director-General has declared seven Public Health Emergencies of International Concern (PHEICs), including 2009 H1N1 swine flu pandemic, 2016 Zika outbreak, the 2014-16 Ebola outbreak, the 2020 COVID-19 pandemic, and the 2022 monkey pox scare. Yet, there are ongoing critiques of the IHR’s limitations to inform governmental responses worldwide [80–82]. Based on the results of this research, we posit the following recommendations:

- Extend research initiatives that examine how countries experience conditional learning from prior outbreaks beyond EVD, as well as the policies that were the most influential
- Build upon and expand large-scale initiatives like the IHR Core Capacity Monitoring Framework technical missions, which proved highly effective among many of the 27 Latin American and Caribbean countries that participated after the EVD outbreak
- Pressure governments to improve their investment in outbreak identification and response programming, primarily targeting nations with recognized policy failures during COVID and other PHEICs
- Improve external funding opportunities such as from the IMF and World Bank to countries that demonstrate the willingness to improve its preparedness but suffer extreme resource-limitations
- Consider increased censorship and economic penalties to governments that do not comply with WHO recommendations during PHEICs
- Consider mandates on annual pandemic preparedness trainings and external reviews in the proposed International Treaty on Pandemic Prevention, Preparedness and Response or Pandemic Treaty, with diverse, inclusive, and equitable consultative expert inputs from nations that have demonstrated considerable learning from post PHEICs.

## Conclusions

Compared to other diseases, Ebola uniquely elicits a macabre fascination among scientist and the general public- be its haemorrhagic nature, onset of grotesque symptoms, high fatality rate, and portrayal in movies and media. There have been dozens of Ebola outbreaks. Yet the 2014-16 West African crisis was the first time Ebola’s spread was transcontinental, affecting tens of thousands of people. These factors likely marked this event as one of the most significant modern-day catalysts for systemic disease response and prevention health reformation worldwide.

This study is the first-known research that attempts to conduct a comparative study of WHO-reporting nation reactions to EVD, and to classify their various responses in relation to COVID. The theoretical framework provided in this report offers a means of measuring the effect of 2014-2016 EVD on nations worldwide. It also offers a map for future research to categorize national responses to specific major pandemic-events, delineated by the institutional and structural changes made by the government as a result of learning from their own direct experiences and/or those of their neighbours. This research, its policy legacies and conditioned learning categories, and related parameters may be modified in future studies. It also offers a foundation to better examine how the effects of biological threats like EVD cause seismic shifts in both the realm of health as in politics and governance.

## Data Availability

The datasets generated and/or analysed during the current study are available in Harvard Dataverse: https://doi.org/10.7910/DVN/APJNDT. A protocol for this systematized review was developed before the research began, is available along with this file.

## Footnotes

1 We determined to include DRC as one of these nations because of the Ebola outbreak in country in July-October 2014. Initially, many experts and media feared that the outbreak was the same Zaire strain from Liberia, Sierra Leone, and Guinea. Later, clinical testing confirmed its zoonotic origin differed from that in the 2014 epidemic in West Africa [43], a fact that garnered little attention. DRC documents additionally state that changes to the national response were based on WHO recommendations from the 2014-16 West African response [7, 43, 44]. Conditioned learning may be affected by countries with a legacy of EVD like DRC and Uganda, and that have close proximity to EVD-affected nations like Burundi. While it may be useful for future research to examine such factors’ statistical influence over conditioned learning categorization, this study prioritizes categorizing nations based on documented evidence of national system changes specifically informed by the 2014-16 EVD outbreak.
